# Causes of reporting bias: a theoretical framework

**DOI:** 10.12688/f1000research.18310.2

**Published:** 2019-07-17

**Authors:** Jenny T van der Steen, Gerben ter Riet, Cornelis A van den Bogert, Lex M Bouter

**Affiliations:** 1 Department of Public Health and Primary Care, Leiden University Medical Center, Hippocratespad 21, Gebouw 3, Leiden, 2300 RC Leiden, The Netherlands; 2 Department of Primary and Community Care, Radboud university medical center, Geert Grooteplein Noord 21, 6500 HB Nijmegen, The Netherlands; 3 ACHIEVE Centre for Applied Research, Amsterdam University of Applied Sciences, Tafelbergweg 51, Amsterdam, 1105 BD Amsterdam, The Netherlands; 4 Department of Cardiology, Amsterdam University Medical Center (location Meibergdreef), University of Amsterdam, Meibergdreef 9, 1105 AZ Amsterdam, The Netherlands; 5 Apotheek Boekel, Kerkstraat 35, Boekel, 5427 BB, The Netherlands; 6 Department of Epidemiology and Biostatistics, Amsterdam University Medical Centers, location VUmc, Van der Boechorststraat 7, 1081 BT Amsterdam, The Netherlands; 7 Department of Philosophy, Faculty of Humanities, Vrije Universiteit Amsterdam, De Boelelaan 1105, 1081 HV Amsterdam, The Netherlands

**Keywords:** Causality, publication bias, questionable research practice, reporting bias, research design, selective reporting

## Abstract

Reporting of research findings is often selective. This threatens the validity of the published body of knowledge if the decision to report depends on the nature of the results. The evidence derived from studies on causes and mechanisms underlying selective reporting may help to avoid or reduce reporting bias. Such research should be guided by a theoretical framework of possible causal pathways that lead to reporting bias. We build upon a classification of determinants of selective reporting that we recently developed in a systematic review of the topic. The resulting theoretical framework features four clusters of causes. There are two clusters of necessary causes: (A) motivations (e.g. a preference for particular findings) and (B) means (e.g. a flexible study design). These two combined represent a sufficient cause for reporting bias to occur. The framework also features two clusters of component causes: (C) conflicts and balancing of interests referring to the individual or the team, and (D) pressures from science and society. The component causes may modify the effect of the necessary causes or may lead to reporting bias mediated through the necessary causes. Our theoretical framework is meant to inspire further research and to create awareness among researchers and end-users of research about reporting bias and its causes.

## Background

### The problem of selective reporting and research on reporting bias

Selective reporting of research findings presents a large-scale problem in science, substantially affecting the validity of the published body of knowledge ( Bouter *et al*., 2016; Dwan *et al*., 2014; van den Bogert *et al*., 2017). Reporting bias (publication bias or outcome reporting bias) occurs when the decision to report depends on the direction or magnitude of the findings. In clinical research, registration of trials prior to data collection is used to prevent selective reporting ( Chan *et al*., 2017; Gopal *et al*., 2018). However, it is insufficiently effective because despite registration or publication of the study protocol, trial results often remain partially or completely unpublished ( Jones *et al*., 2013) and selective reporting of “positive findings” also occurs among trials registered at, for example, clinicaltrials.gov ( Dechartres *et al*., 2016).

 Although many epidemiological studies have described the occurrence or phenomenon of selective reporting, very few studies have targeted its causes. In particular there is little high-quality evidence on effective interventions. To develop effective interventions against reporting bias, we need a good understanding of possible contributions of actors involved (such as academic environment, editors, researchers) and of possible mechanisms. We also need clear hypotheses of how causes may be interrelated.

### Basis for a theoretical causal framework: hypothesized determinants of selective reporting and their interrelationships

We recently developed a taxonomy of putative determinants of selective reporting based on themes abstracted from the literature ( van der Steen *et al.*, 2018). We used an inductive approach of qualitative content analyses of empirical and non-empirical studies until we reached saturation, which indicates that the categories likely cover all important putative determinants of selective reporting. This resulted in 12 categories ( Table 1).

**Table 1.** Twelve categories of determinants of selective reporting. (Modified from the taxonomy of determinants presented in Table 3 in: Determinants of selective reporting: A taxonomy based on content analysis of a random selection of the literature. van der Steen JT *et al.* PLoS One. 2018 Feb 5;13(2):e0188247. doi: 10.1371/journal.pone.0188247.)

Determinant category	Description	Examples
**A. Motivations**		
Preference for particular findings	A particular preference motivates a focus on finding results that match preferences, mostly statistically significant or otherwise positive findings, wishful thinking and acting	Significance chasing, finding significant results, larger effect size, suppressing publication of unfavourable results, not being intrigued by null findings
Prejudice (belief)	A conscious or unconscious belief that may be unfounded, and of which one may or may not be aware	Prior belief about efficacy of treatment, author reputation or gender bias in the phase of review
**B. Means**		
Opportunities through poor or flexible study design *	Attributes of study design relating to power and level of evidence provide much leeway in how studies are performed and in interpretation of their results	Not a controlled or blinded study, study protocol unavailable, small sample size
Limitations in reporting and editorial practices	Constraints and barriers to the practice of reporting relevant detail	Journal space restrictions, author writing skills
**C. Conflicts and balancing of interests**		
Relationship and collaboration issues	Intellectual conflict of interest between reporting and maintaining good relationships	Disagreements among co-authors and between authors and sponsors, sponsors prefer to work with investigators who share the sponsor’s position
Dependence upon sponsors	Financial conflict of interest resulting in lack of academic freedom	Requirements and influence of funding source with financial interests in study results
Doubts about reporting being worth the effort	Weighing investment of time and means versus likelihood of gain through publication	Anticipating disappointment of yet another rejection or low chances of acceptance of a manuscript, belief that findings are not worth the trouble
Lack of resources, including time	Insufficient manpower or finances	Lack of time resulting from excessive workload, or lack of personnel due to life events
**D. Pressures from science and society**		
Academic publication system hurdles	Various hurdles to full reporting related to submission and processing of manuscripts (other than reporting) including those that represent an intellectual conflict of interest	Solicited manuscripts, authors indicating non- preferred reviewers, editor’s rejection rate
High-risk area and its development	Area of research or discipline or specialty including its historical development and competitiveness, the currently dominant paradigms and designs, and career opportunities	Ideological biases in a research field, area with much epidemiological research versus clinical or laboratory research (“hard sciences”), humanities, experimental analytic methods, “hot” fields, publication pressure in the specific field
Unfavourable geographical or regulatory environment	Geographical or regulatory environment that affects how research is being performed	Continents under study included North America, Europe and Asia; few international collaborations; no governmental regulation of commercially sponsored research, ethics in publishing enterprise
Potential harm	Publishing data can harm individuals	Risk of bioterrorism, or confidentiality restriction

^*^With study design, we mean broader design issues than just type of research design, including also definitions, outcomes, analytic plans etc.

In the literature review we also found some instances of hypothesized effect modification of the determinants of selective reporting, so that the effects of determinants are assumed not to be simply additive. For example, “Outcomes could be deemed post hoc to have little clinical relevance if they fail to show significant findings and may thus be omitted *when* accommodating space limitations” ( Chan & Altman, 2005). In this case, a preference, namely statistically significant findings, combined with editorial practices lead to reporting bias. Similarly, Ioannidis (2005) hypothesized that a focus on preferred, positive findings could result in reporting of non-reproducible findings (only) if there is also an opportunity to do so through flexibility in study designs and freedom in reporting on it. That is, he concludes that “The greater the flexibility in designs, definitions, outcomes, and analytical modes in a scientific field, the less likely the research findings are to be true” because “Flexibility *increases the potential* for transforming what would be ‘negative’ results into ‘positive’ results.”

## A framework of possible causal pathways to reporting bias

### Motivations and means

**Figure 1.**
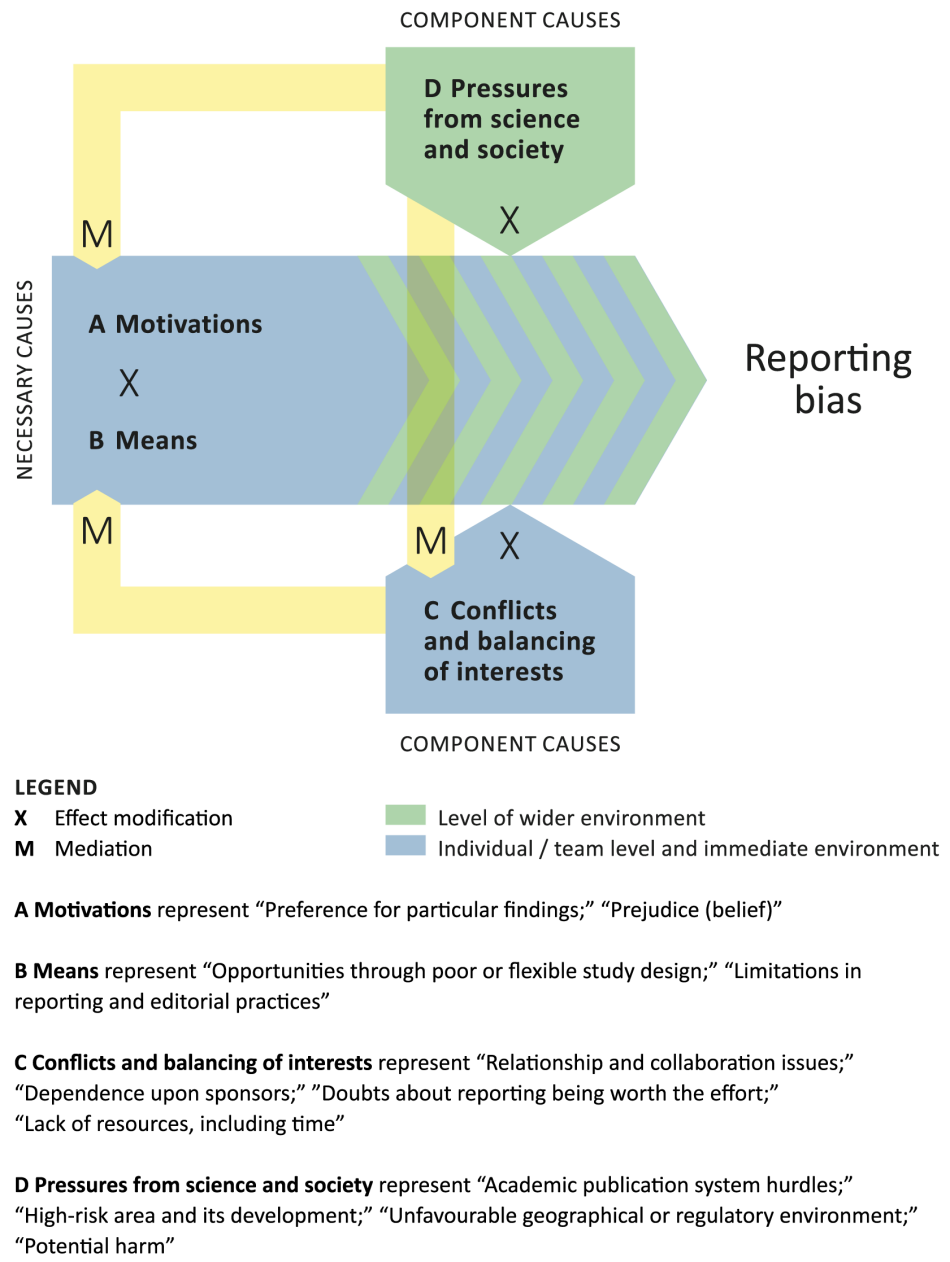
A theoretical framework for reporting bias. Bullet points indicate the 12 categories of determinants of selective reporting subsumed under four higher-level clusters A, B, C, and D. Note that the figure implies effect modification between A and B (necessary causes) because there will be no reporting bias with A or B alone. Effect modification (“X”) may also occur by C or D and thus make the joint effect of A and B stronger. Mediation (“M”) may occur if the necessary causes (A and B) mediate the effect of D. Mediation may also occur if C mediates the effects of D on A and B, which in its turn leads to reporting bias.

Based on what we found in the literature and along the above lines, we hypothesize that the combination of two of the most common categories in our review ( van der Steen *et al*., 2018) –– i.e., focusing on preferred findings and employing a poor or flexible study design, suffices to cause bias through selective reporting. Through multiple discussions in our team featuring experience in both qualitative and quantitative research, we inductively derived Figure 1 which shows how, as a next step, we identified and presented clusters covering these and the ten other categories of determinants and their possible interrelationships. We then added qualifications of the relationship inspired by Rothman’s (1976) framework of necessary, sufficient and component causes. The two categories are part of clusters A (motivations) and B (means). We view both clusters A and B as necessary causes, that is, they are both part of any sufficient cause of reporting bias. This does not mean that reporting bias will always be the result of presence of A and B because effects can be mitigated by interventions and modified by component causes. Applying more epidemiological terms to the generic model we developed, there is also effect modification between A and B because reporting bias is not possible with A or B alone. Note that a preference for a particular outcome is not necessarily the authors’ preference; it may also be that of a reviewer or editor. In addition to clusters A and B, we propose clusters C and D containing categories of component causes which are discussed in the next section.

Poor or flexible study design may offer the means for selective reporting in addition to limitations in reporting and editorial practices (cluster B in Figure 1). In parallel, we placed “prejudice” in cluster A together with “preference for particular findings” because both may, whether consciously or not, represent a motivation for behaviour that leads to reporting bias. The possible motivations, wishes and beliefs in cluster A are different concepts that may result in “wishful thinking” ( Bastardi *et al*., 2011) and in motivated reasoning around the interpretation of scientific findings (e.g. to serve political interests; Colombo *et al*., 2016; Kraft *et al*., 2015). Persons may or may not be fully aware of their motivations and the resulting behaviour may or may not be intentional ( Greenland, 2009). Dickersin & Min (1993) stated that at the root of reporting bias may lay the very natural tendency to make public our successes. Success can be defined in different, or even opposite ways as suggested by Rosenthal and Rubin cited by Preston *et al*. (2004) whose article was part of our review: “[E]arly in the history of a research domain results in either direction are important news but that later, when the preponderance of evidence has supported one direction, significant reversals are often more important news than further replications.”

The pertinence of the second necessary cause (cluster B)––multiple opportunities to select what to analyse or report––is illustrated by the many degrees of freedom that researchers have but should not be tempted to use (in performing psychological research: Wicherts *et al*., 2016). The necessary causes thus represent having a motive (preference or prejudice; cluster A) and the means (opportunities in study design or reporting; cluster B). Together they may form a sufficient cause for reporting bias.

Obviously, researchers and editors are key stakeholders because commonly they co-determine what will be reported. It can be argued that researchers are the most important because a single editor’s decision is not decisive for non-publication or selective publication. Researchers are actors in three of the four categories in clusters A and B that represent the necessary causes, while editors are key players in only one category (in cluster B; Figure 1). Note that we assume actors in the field are capable of effective action.

### Conflicts and balancing of interests and the wider environment

In the review, we found that after a series of rejections researchers may doubt whether reporting is worth the effort given lack of resources such as time. Balancing effort and output is placed in cluster C (component cause conflicts and balancing of interests; Figure 1). Cluster C also includes relationship and collaboration issues and dependence upon sponsors. Cluster C thus represents conflicts of interests, individuals and teams juggling with harmony in relationships and time investments.

Other component causes represent pressures from the wider environment, such as from science and society (cluster D). The individual researcher has less control over type C, and in particular type D causes, than over motivations (A) and means (B). C and D cannot fully control or explain individuals’ decisions, but they may shape motivations (A) and means (B). When this is the case the effect on reporting bias of the categories in cluster C or D is mediated through the categories contained in cluster A or B. For example, important news is selectively reported but what is deemed important news is shaped by the development within a scientific domain (cluster C; Preston *et al*., 2004). Also, researchers’ collaborations or relations with sponsors may nudge them to selectively report the preferences of others. A final example is academic publication system hurdles (cluster D) and dependence upon sponsors (cluster C) leading to reporting bias through their impact on the combination of a preference for positive findings and the opportunities that flexible designs offer.

## Discussion

We propose a broad theoretical framework of reporting bias by relating and ordering 12 determinant categories that we derived from the literature ( van der Steen *et al*., 2018). We inductively combined these categories in four clusters (A–D) using existing epidemiologic terminology to label relationships.

The model is more refined than we anticipated when we wrote a protocol to develop a taxonomy of determinants of selective reporting and their interrelationships. We then expected a central role for preferences for particular “positive” findings only ( van der Steen *et al*., 2018 Supplement 1, Figure 1). However, having the means is necessary too. Although the determinants in our model are mostly based on research in the biomedical area, the model fits well with the “Desire-Belief-Opportunity” (DBO) model that analytical sociologists use to explain various phenomena ( Hedström, 2005) and which we came across after having developed our theoretical framework. Desire and Belief concur with the two motivations in cluster A, while opportunities (alternative actions available to the actor) represent the means in cluster B.

Theory may guide the development of interventions as research often does not systematically consider contextual and individual factors that influence delivery of an intervention. Thus, theory may help avoid an ad hoc or data-driven approach to attempts to reduce reporting bias. It may also help explain some other phenomena, for example, problems with replicability which are partly caused by selective reporting. Replication studies can effect the four clusters A–D. They can impact on Motivations when e.g., researchers more often aim at study results that are likely replicated, or when researchers conducting replication studies are more open to, or working towards, null results. They can also impact on Means, e.g. the rise of specific journals that support publishing replicated studies, and on Conflicts and balancing of interests (e.g. earmarked resources for replication studies becoming available), and Pressures from science and society (e.g. less creative and innovative but rigorous research becoming more salonfähig).

Although one might assume that interventions addressing reporting bias effectively will be complex, the removal of a single necessary cause is obviously effective. For example, a potentially very effective measure that funders and (medical) ethics committees could adopt is systematic monitoring of all written research outputs and comparing the outcomes reported therein to the corresponding research protocols and statistical analysis plans and potential amendments. This would require that these organizations make submission of such documents to them or to a publicly available repositories mandatory, in addition to requiring submission of a research protocol or study registration. For this, automated or manual comparing protocols to publications is needed ( ter Riet & Bouter, 2016; Wright *et al.*, 2018). In the jargon of this paper, this approach would eliminate the necessary cause ‘Means.’ Given suitable negative reinforcements (punishments, ‘blacklist’) following incomplete reporting, such measures may also reduce motivation to report selectively. Similarly, elements from the component causes contained in cluster C and D that are highly prevalent and strongly modify the combined effect of cluster A and B may be prioritized targets. Mediators can also be good candidates for intervention. For example, component causes contained in cluster C may mediate the impact of elements of D on elements of clusters A or B. The model may also assist in assessing potential confounding factors in observational work in which associations between a specific determinants and reporting bias is assessed.

In addition to informing the development of interventions that are subsequently evaluated, our framework may also help to identify high risk scientific fields. For example, areas where designs offer considerable flexibility or where the researchers’ degrees of freedom are combined with strong beliefs or a mission to disseminate particular outcomes ( Ioannidis, 2005). The model also shows that for example editors may influence the outcome in multiple ways; first, directly via Means (the category Limitations in reporting and editorial practices). Second, editors as a collective affect the rejection rates through Academic publication system hurdles (the category of Pressures from science and society), but also the extent to which authors find efforts to publish worthwhile (category of cluster Conflicts and balancing of interests). The latter is illustrated by the personal account of Speyer (2018).

Currently, the evidence for the theoretical framework is limited. Based on research, our theoretical framework may need to be adapted. Motive and Means may be stable clusters but the C and D type causes may change as science changes. Future work may also help to refine the framework’s relevance for specific disciplinary fields (e.g., non-clinical biomedical research). Further empirical research is needed to specify, for example, what could be an optimal level of flexibility for a particular field and study design. Nevertheless, because the causal pathways seem plausible, were derived from the literature on selective reporting and is congruent with theory developed in the social sciences ( Hedström, 2005), we feel that the current work can already help to design further research on the effectiveness of interventions.

## Data availability

### Underlying data

PLOS ONE Supplement 2 to article van der Steen *et al*., 2018. Determinants of selective reporting abstracted from the selected literature. “S2 File. Dataset with determinants.” In Excel available from: https://doi.org/10.1371/journal.pone.0188247.s003 ( van der Steen *et al*., 2018)

PLOS ONE Supplement 3 to article van der Steen *et al*., 2018. Categories of determinants of selective reporting with literature references. “S3 file. References to the 64 articles included in the determinant analysis, per category.” In Word available from: https://doi.org/10.1371/journal.pone.0188247.s004 ( van der Steen *et al*., 2018)

Data are available under the terms of the Creative Commons Attribution 4.0 International license (CC-BY 4.0).
