# Supplementary material for: Causes of reporting bias: a theoretical framework
Source: F1000Res. 2019 Jul 17;8:280. Originally published 2019 Mar 12. [Version 2] doi: 10.12688/f1000research.18310.2 (PMC6713068; doi:10.12688/f1000research.18310.2)
Supplement: Peer review history

# Peer review: Reviewer response for version 2

**DOI:** 10.5256/f1000research.21747.r51330

**Published:** 2019-08-27

**Authors:** Arnaud Vaganay

**Affiliations:** 1 Meta-Lab, London, UK; 2 National Centre for Social Research (NatCen), London, UK

I thank the authors for their response to my comments and for addressing my concerns/suggestions. 

 I am now satisfied with the article and leave it to the reader to assess the framework for themselves. 

 I hope this article will trigger more research on this fascinating subject.

I confirm that I have read this submission and believe that I have an appropriate level of expertise to confirm that it is of an acceptable scientific standard.

# Peer review: Reviewer response for version 2

**DOI:** 10.5256/f1000research.21747.r51331

**Published:** 2019-08-14

**Authors:** Ksenija Bazdaric

**Affiliations:** 1 Department of Medical Informatics, Faculty of Medicine, University of Rijeka, Rijeka, Croatia

I approve the manuscript. The model is a specific one, does not include some factors which I have mentioned in my first report and still has to be tested.

 I am happy with the revisions you have done. 

 Good luck in your endeavours.

I confirm that I have read this submission and believe that I have an appropriate level of expertise to confirm that it is of an acceptable scientific standard.

# Peer review: Reviewer response for version 1

**DOI:** 10.5256/f1000research.20029.r47229

**Published:** 2019-05-20

**Authors:** Arnaud Vaganay

**Affiliations:** 1 Meta-Lab, London, UK; 2 National Centre for Social Research (NatCen), London, UK

I was pleased to review this manuscript. I assessed the proposed theoretical framework based on the following criteria: (1) utility, (2) comprehensiveness, (3) parsimony, (4) testability, (5) heurism, and (6) scope. Ironically, these criteria are not part of an established theoretical framework; they only reflect a brief review of the literature on the subject.

 I would rate the utility of the proposed framework as high. As far as I know, this is one of the first attempts to synthesize the literature on the factors driving reporting bias. Additionally, and as mentioned by the authors, understanding these factors is essential to the development of mitigating measures.

 I would rate the heurism (i.e. evidence base) of the framework as low. Granted, there is now a large body of literature on the prevalence of reporting bias and on the possible factors driving it. However, virtually none of these studies are experimental, so all the relationships found between the occurrence of reporting bias and the 12 categories are at best correlational – not causal. This is, in my view, the most fundamental flaw of the framework. The good news is, it could be easily addressed by changing the title of the manuscript from “Causes of reporting bias” to “Drivers of reporting bias”. 

 Related to the above-mentioned point, I would rate the testability of the framework as low. The only testable driver of reporting bias is “financial conflict of interests” (FCOI). Most other drivers (prejudice, relationship and collaboration issues, doubts about reporting being worth the effort, etc.) would be hard to test empirically. Virtually none of these drivers can be tested in experimental conditions. 

 I was unable to rate the comprehensiveness of the framework for three reasons. Firstly, and as already mentioned, I do not think that the proposed framework can realistically identify the possible “causes” of reporting bias – let alone all the possible causes. Secondly, the authors constructed the typology based on a “systematic review”, which I haven’t seen. Systematic reviews of narrowly defined questions are notoriously hard to conduct; I don’t think that a review of such a broad question can be truly systematic. Thirdly, I don’t have enough knowledge of the topic to suggest missing/alternative categories.

 I would rate the parsimony of the framework as medium. On the one hand, I do not think it is possible to propose a parsimonious framework when the scope of the theory is so broad. On the other hand, Table 1 is much more parsimonious than the vast literature it draws on.

 I would rate the scope of the framework as fairly high. The authors acknowledge that the determinants of the model “are mostly based on research in the biomedical area”. However, I would argue that the 12 categories listed in the framework are also relevant to social research, which is the type of research that I do myself. 

 In conclusion, I am grateful for the authors’ contribution to the literature and do believe that the proposed framework could help the research community address the problem of reporting bias. However, I have some concerns regarding (i) the strength of the evidence used by the authors to make causal claims; and (ii) the testability of the framework.

I confirm that I have read this submission and believe that I have an appropriate level of expertise to confirm that it is of an acceptable scientific standard, however I have significant reservations, as outlined above.

# Response

**Published:** 2019-06-26

**Authors:** Jenny van der Steen

**Affiliations:** Leiden University Medical Center, The Netherlands

1. I was pleased to review this manuscript. I assessed the proposed theoretical framework based on the following criteria: (1) utility, (2) comprehensiveness, (3) parsimony, (4) testability, (5) heurism, and (6) scope. Ironically, these criteria are not part of an established theoretical framework; they only reflect a brief review of the literature on the subject.

**Response**: Thank you. We appreciate the reviewing of our work against a series of relevant criteria.

2. I would rate the utility of the proposed framework as high. As far as I know, this is one of the first attempts to synthesize the literature on the factors driving reporting bias. Additionally, and as mentioned by the authors, understanding these factors is essential to the development of mitigating measures.

**Response**: Thank you, indeed we aimed at identifying candidate determinants of reporting bias and interrelationships with a view to develop interventions in a more systematic way, and to allow for testing to improve the theoretical framework.

3. I would rate the heurism (i.e. evidence base) of the framework as low. Granted, there is now a large body of literature on the prevalence of reporting bias and on the possible factors driving it. However, virtually none of these studies are experimental, so all the relationships found between the occurrence of reporting bias and the 12 categories are at best correlational – not causal. This is, in my view, the most fundamental flaw of the framework. The good news is, it could be easily addressed by changing the title of the manuscript from “Causes of reporting bias” to “Drivers of reporting bias”. 

**Response**: We fully agree that the evidence base for the theoretical framework is still low. We found only 1 randomized trial in the 64 articles we analysed in the review. Numerous studies have been performed, and also interventions such as training and pre-registration have been implemented but very few interventions have been evaluated thoroughly. This is why we speak about putative determinants and plausible mechanisms. We are not sure whether “drivers” is essentially different from “causes.” Heurism is probably in how we could learn about causes. To this end, it is important to consider the subtitle which is “a theoretical framework.” This can be developed also on the basis of a modest evidence base, because the aim is to test the model’s assumptions and adapt the model if necessary. This might progress the field at a faster pace than continuing without any or only an implicit theoretical framework. At least, we believe that considering the current evidence base, developing a theoretical framework is timely. We do recognize the small evidence base of the framework.

*To the last paragraph of the Discussion, we added a sentence, to start the paragraph with explicitly recognizing the small evidence base: “Currently, the evidence for the theoretical framework is limited.”*

4. Related to the above-mentioned point, I would rate the testability of the framework as low. The only testable driver of reporting bias is “financial conflict of interests” (FCOI). Most other drivers (prejudice, relationship and collaboration issues, doubts about reporting being worth the effort, etc.) would be hard to test empirically. Virtually none of these drivers can be tested in experimental conditions. 

**Response**: We agree that testing the model will require thoughtful consideration of putative determinants and how these could be manipulated. Models can be helpful even if not testable in a traditional way and many theories have been developed and maintained this way. Further, we need to consider how other than experimental designs can facilitate a better understanding of causes (importantly, more in-depth and thorough qualitative research which has been largely neglected in this area). The model itself shows that the issue is multifactorial which means often multicomponent interventions are required which complicates identifying contributions of single components. Creative experimental designs such as manipulating realistic scenarios withholding or adding information in different conditions and choice experiments may offer alternative ways of researching researchers in situations that are otherwise standardized. We agree that testing the model will require huge efforts, but we do not believe it is impossible.

Perhaps the focus of the Discussion was too much on experimental work. The model may also improve the quality of design of observational studies. For example, when, to assess associations with a specific determinants, the model inspires to measure important possible confounding factors.

*We added to the Discussion* *: “The model may also assist in assessing potential confounding factors in observational work in which associations between a specific determinant and reporting bias is assessed.”*

5. I was unable to rate the comprehensiveness of the framework for three reasons. Firstly, and as already mentioned, I do not think that the proposed framework can realistically identify the possible “causes” of reporting bias – let alone all the possible causes. Secondly, the authors constructed the typology based on a “systematic review”, which I haven’t seen. Systematic reviews of narrowly defined questions are notoriously hard to conduct; I don’t think that a review of such a broad question can be truly systematic. Thirdly, I don’t have enough knowledge of the topic to suggest missing/alternative categories.

**Response**: Our systematic review was published in PLOS One (2018). We apologize that we had referred to it only in the section explaining the model and not yet in the Background section. The search was systematic, but we analysed only a random sample of the literature because indeed the broad question did not allow to analyze all relevant literature. However, we assessed saturation of the qualitative content analyses of determinants abstracted from the literature in two ways, prospectively and retrospectively, both of which indicated that finding additional categories of determinants through analyzing more articles was unlikely. We were interested in the main categories which cover a number of specific determinants which may also differ somewhat for different disciplines. We commented on possible refinement needed for specific disciplinary fields in the Discussion section.

*We added explicit reference to the review, inserting it after* ”We recently developed a taxonomy of putative determinants of selective reporting abstracted from the literature” *( van der Steen et al., 2018). Next, we clarified the methods by several additions to the paragraph of “A framework of possible causal pathways to reporting bias.”*

6. I would rate the parsimony of the framework as medium. On the one hand, I do not think it is possible to propose a parsimonious framework when the scope of the theory is so broad. On the other hand, Table 1 is much more parsimonious than the vast literature it draws on.

**Response**: Thank you. In the qualitative content analyses of the literature we aimed at grouping together related determinants.

7. I would rate the scope of the framework as fairly high. The authors acknowledge that the determinants of the model “are mostly based on research in the biomedical area”. However, I would argue that the 12 categories listed in the framework are also relevant to social research, which is the type of research that I do myself. 

**Response**: Thank you. The literature we analysed predominantly concerned clinical medicine and biomedicine, but the humanities represented 11% of articles. The model also resembles the Desire-Belief-Opportunity” (DBO) model from sociology.

8. In conclusion, I am grateful for the authors’ contribution to the literature and do believe that the proposed framework could help the research community address the problem of reporting bias. However, I have some concerns regarding (i) the strength of the evidence used by the authors to make causal claims; and (ii) the testability of the framework.

**Response**: We hope that our theory about causality which clearly cannot reach farther than the evidence base and literature so far, inspires researchers to develop smart designs to discover causal mechanisms and to propose adaptation of the theory if needed. Thank you for this interesting and thoughtful input.

*Other changes.*

*We edited the text at several places, shortening it where possible.*

*In the Discussion, we shortened the example of an intervention with regard to the means for comparing protocols and publication. Software to compare protocol and publication was not necessary in the NIHR Journal Library system and we added reference to Wright et al. (2018) for this*.

# Peer review: Reviewer response for version 1

**DOI:** 10.5256/f1000research.20029.r46634

**Published:** 2019-04-11

**Authors:** Ksenija Bazdaric

**Affiliations:** 1 Department of Medical Informatics, Faculty of Medicine, University of Rijeka, Rijeka, Croatia

I was happy to review a manuscript about a theoretical framework in the field of reporting bias. I think the authors have proposed an interesting perspective but my major remark is that they try to explain human behaviour with an epidemiological model for which I don’t find a body of evidence in the literature that could convince me. 

 Comments: 

1. Background: In clinical research, registration of trials prior to data collection is used to prevent selective reporting with some success – please delete “some success” because it is further explained.
2. " **A framework of possible causal pathways to reporting bias - Motivations and means.**
  Along these lines, we hypothesize that the combination of two of the most common categories in our review (van der Steen et al., 2018) –– i.e., focusing on preferred findings and employing a poor or flexible study design, suffices to cause bias through selective reporting..." – how do you then comment on the replication crisis in psychology and the experiments that were replicated in the same laboratories? They were motivated to replicate and for sure were not sloppy. Do we have a poor designed field here or are there other factors? I would like a more detailed explanation ^1^^,^^2^^,^^3^.
3. A theoretical framework for reporting bias. Rothman’s theoretical model – is there any evidence in practice for this model in relation to human behaviour? 
  Figure 1  and the model: It is an interesting figure, but the same could be explained by some other general theories, for example  'Theory of planned behaviour' (of course evidence cannot be confirmed as we have a replication crisis in psychology). I don’t believe human behaviour can be explained with an epidemiological model although it is very nice. Also, the model itself does not have a word about ethical climate and other possible external factors. Why did you exclude them? Do you consider them stable in all environments?
  There is some evidence that peers can predict replication ^2^? How do you comment? Could you include some external factors in your model? Like environment, ethical climate, etc…
4. The sentence: "At the root of reporting bias may thus lay a basic human attitude, the very natural tendency to make public our successes" – this is not clear at all. At a root of everything probably lies personality and attitude, but I don’t understand the meaning of the sentence here.
5. Obviously, researchers and editors are key stakeholders because commonly they decide what is actually being reported and what is not. – I would say that sometimes we cannot report everything because we have only 3000-4000 words (here is the role of editors).
6. After a series of rejections researchers may doubt whether reporting is worth the effort under the pressure of lack of resources such as time. I advise you to read a case study from Helene Speyer - The value of a “failed” trial ^4^.

I confirm that I have read this submission and believe that I have an appropriate level of expertise to confirm that it is of an acceptable scientific standard, however I have significant reservations, as outlined above.

## References

[1] : 1,500 scientists lift the lid on reproducibility. *Nature News*.2016; Reference source 10.1038/533452a27225100

[2] : Evaluating the replicability of social science experiments in Nature and Science between 2010 and 2015. *Nature Human Behaviour*.2018;2(9) : 10.1038/s41562-018-0399-z 637-644 10.1038/s41562-018-0399-z 31346273

[3] : PSYCHOLOGY. Estimating the reproducibility of psychological science. *Science*.2015;349(6251) : 10.1126/science.aac4716 aac4716 10.1126/science.aac4716 26315443

[4] : Discovering the value of a “failed” trial. *European Association of Science Editors*.2018;44(4) :80-82

# Response

**Published:** 2019-06-26

**Authors:** Jenny van der Steen

**Affiliations:** Leiden University Medical Center, The Netherlands

I was happy to review a manuscript about a theoretical framework in the field of reporting bias. I think the authors have proposed an interesting perspective but my major remark is that they try to explain human behaviour with an epidemiological model for which I don’t find a body of evidence in the literature that could convince me. 

**Reponse**: Thank you so much for reviewing our manuscript and considering it from the perspective of research in humanities.Thank you also for your interesting viewpoint regarding types of models, which is helpful to clarify the background of our model.

We respectfully disagree that qualifications such as *epidemiological model*, or *behavioural model* apply well to our model. The model has been developed from determinants of selective reporting mostly in the medical and biomedical literature, but we also retrieved determinants from, e.g., the humanities (11% of articles, van der Steen *et al*., PLOS One 2018). We analyzed the determinants inductively using qualitative content analyses. This means we did not impose any previous model when we identified and categorized the determinants. Texts from the original articles inspired us to consider how determinants might work together, also distinguishing between clusters of related determinants. In a next step, we indeed recognized and used epidemiological terms to label relationships between categories of determinants in terms of effect modification and interaction. Text in the original articles also inspired us to consider whether some categories of determinants could be necessary causes, which is also terminology used in epidemiology.

However, we could just not use the terms and arrive at the same model inspired by the literature about how categories of determinants could work together. We feel that using certain terminology does not suffice to qualify the model in such a way that it limits application to fields that do not use that terminology. The essence is our inductive way of forming categories and in part, also the thinking of relationships has been taken from the literature using an open approach about how categories may work together.

Moreover, the outcome is reporting bias in the literature which is very broad. It cannot be brought about by the behavior of a single individual. Therefore, we do not claim we can predict individual behavior. In contrast, the core of our model ¾ the necessary causes that indeed refer the most to behavior of individuals ¾ probably resembles the most a broad model from sociology (“Desire-Belief-Opportunity,” DBO), which we found by coincidence after having conceived our model. Finally, the cluster of Pressures from science and society comprises four societal determinants of reporting bias and we visualized it can impact behavior of individuals directly but also indirectly.

In all, we believe there is little reason to qualify the model as belonging to a single particular discipline imposing disciplinary boundaries around its application. We rather hope that our model will cross such boundaries and help researchers from different disciplines and perhaps also policy makers to work together to address reporting bias.

*In the Background paragraph of “Basis for a theoretical causal framework: hypothesized determinants of selective reporting and their interrelationships” we added that we had used aninductive approach to develop the *categories *. Further, we connected the paragraph with the next paragraph (“A framework of possible causal pathways to reporting bias”) more clearly by starting it with “Based on what we found in the literature.” Next, we reversed the order in the sentence about how we applied Rothman’s classification of causes, because indeed we first agreed on the relationships and we used Rothman’s labels to express the relationships in terms of his nomenclature. The sentence that started with “Inspired by Rothman’s (1976) framework of necessary, sufficient and component causes,” now starts with “Through multiple discussions, we inductively derived Figure 1.” In the following sentence, we also clarified that we applied terms from epidemiology to what we found in an inductive way, by starting it with: “Applying more epidemiological terms to the generic model we developed,”*

*We reflect on the approach and background of the authors by adding to the same paragraph that the discussions were “ among the team with experience in both qualitative and quantitative research.” Finally, to the first sentence of the Discussion, we added that our theory is “ broad” and that we combined categories into clusters “inductively”, “using existing epidemiologic terminology to label relationships.”*

Comments: 

1. Background: In clinical research, registration of trials prior to data collection is used to prevent selective reporting with some success – please delete “some success” because it is further explained.

**Response**: *We deleted as suggested “some success.”*

2. " **A framework of possible causal pathways to reporting bias - Motivations and means.**

Along these lines, we hypothesize that the combination of two of the most common categories in our review (van der Steen *et al*., 2018) –– i.e., focusing on preferred findings and employing a poor or flexible study design, suffices to cause bias through selective reporting..." – how do you then comment on the replication crisis in psychology and the experiments that were replicated in the same laboratories? They were motivated to replicate and for sure were not sloppy. Do we have a poor designed field here or are there other factors? I would like a more detailed explanation ^1^ ^,^ ^2^ ^,^ ^3^.

**Response**: Thank you for your comment. The interesting Nature survey on non-reproducibility that you cited (Baker, 2016) ^1^ indicates that researchers believe selective reporting is a major contributor to research often not being replicable, and also a number of factors which in our model are determinants of reporting bias (e.g., pressure to publish and poor experimental design). Of course the Nature survey did not aim at offering a model or mechanisms and therefore outcomes and determinants are not being distinguished. Despite probably sharing determinants, the survey also includes “methods, code unavailable” as a reason for non-reproducibility which underlines that reporting bias is not the only reason that research is not reproducible.

Further, perhaps it was not clear enough that the model does not assert that if means and motivations are there, these are sufficient causes but this will *not necessarily* result in reporting bias. The model includes Conflicts and balancing of interest and Pressures from science and society which can modify effects on reporting bias. Further, not included in our model are interventions. Interventions may alter motivations or change the flexibility of designs and therefore diminish effects on reporting bias. We hope the model will inspire development of targeted interventions.

*After “We view both clusters A and B as necessary causes, that is, they are both part of any sufficient cause of reporting bias” we added: “This does not mean that reporting bias will always be the result of presence of A and B because effects can be mitigated by interventions and modified by component causes.” Further, we added in this paragraph the term “may” in: “Together they may form a sufficient cause for reporting bias.”*

The model does not specify what kind of motivations researchers may have, what would be the most interesting outcome in a particular case (e.g., in our review we found motivations to try to find the same as other research early in development of a field, but motivations later on changed to find deviations). Also we do not provide criteria for how much or how little flexibility in design would be optimal or qualify particular research as sloppy.

*We further extended the paragraph with a citation from our review about motivation to report news and how the contents of the news may vary over time, in addition to citing it later on referring to development of a field: “Success can be defined in different, or even opposite ways as suggested by Rosenthal and Rubin cited by Preston et al. (2004) which was part of our review:  “early in the history of a research domain results in either direction are important news but that later, when the preponderance of evidence has supported one direction, significant reversals are often more important news than further replications.” This example also illustrates the intertwining of individual motivations and societal factors.*

Flexibility of design may actually be an advantage in some cases as long as the reporting about the methods is transparent, and some designs are flexible by nature. Such specifications would require empirical research.

*To the last paragraph of the Discussion, we added: “Further empirical research is needed to specify, for example, what the optimal level of flexibility for a particular field and study design would be.”*

3. A theoretical framework for reporting bias. Rothman’s theoretical model – is there any evidence in practice for this model in relation to human behaviour? 

Figure 1  and the model: It is an interesting figure, but the same could be explained by some other general theories, for example  'Theory of planned behaviour' (of course evidence cannot be confirmed as we have a replication crisis in psychology). I don’t believe human behaviour can be explained with an epidemiological model although it is very nice. Also, the model itself does not have a word about ethical climate and other possible external factors. Why did you exclude them? Do you consider them stable in all environments?

There is some evidence that peers can predict replication ^2^? How do you comment? Could you include some external factors in your model? Like environment, ethical climate, etc…

**Response**: As explained above in our response to the general comment, Rothman’s terms were applied later on, when the categories of determinants were already retrieved in an inductive manner from literature about determinants of selective reporting. We believe that the theory of planned behavior is not very suitable to explain reporting bias as a phenomenon based on behavior of multiple individuals, teams, institutes, peer groups, or society. How would it incorporate the categories we found in the literature, such as “Unfavourable geographical or regulatory environment” (Table 1)? Further, various other theories may apply to parts of our model, for example, from criminology, theories about strain and goal displacement.

Reviewing our dataset with 497 determinants, we find, for example, one of the determinants from the literature classified under this heading was “Increased accountability from medical journals regarding ethics and conflicts of interest” (Dieterich *et al*., 2013; Supplemental file 2 to the review in PLOS One). It relates to the ethical climate in the publishing enterprise. Of note, at this point, we would not revise categories that are not being supported by the content analysis of the literature, but we can clarify the existing categories by providing more examples.

*To further clarify the category of  Unfavourable geographical or regulatory environment”  in Table 1, we added the example of “ethics in publishing enterprise”*

Replication studies can help define the scope of problems with replicability which may be due to reporting bias only in part. Replication studies can also affect the four clusters A-D. It can impact Motivations (e.g., researchers becoming more interested in study results that are likely replicated or researchers conducting replication studies being more open to, or working towards in null results), Means (e.g. rise of specific journals that support publishing replicated studies), Conflicts and balancing of interests (e.g. earmarked resources for replication studies becoming available), and Pressures from science and society (e.g. less creative and innovative but rigorous research becoming more salonfähig). *We added this example of replication studies impacting clusters A-D to the Discussion.*

4. The sentence: "At the root of reporting bias may thus lay a basic human attitude, the very natural tendency to make public our successes" – this is not clear at all. At a root of everything probably lies personality and attitude, but I don’t understand the meaning of the sentence here.

**Response**: we cited from an article included in the review in PLOS One because those articles provided the data we analysed to develop the determinant categories. Perhaps the reasoning was not clear enough.

*Although we would not repeat the methods of the review in full, we added explicit reference to the review earlier in the text, inserting it in the second paragraph of the Background, after ”We recently developed a taxonomy of putative determinants of selective reporting abstracted from the literature” (van der Steen et al., 2018).*

Further, the authors of the statement find a general attitude to show success relevant for reporting bias. They do not claim to explain all human behavior. We feel the emphasis about showing success is a key issue. *We deleted “a basic human attitude” to avoid confusion about the key point.*

5. Obviously, researchers and editors are key stakeholders because commonly they decide what is actually being reported and what is not. – I would say that sometimes we cannot report everything because we have only 3000-4000 words (here is the role of editors).

**Response**: We agree. We found this in several articles. Table 1 therefore shows that one of twelve categories of determinants was “Limitations in reporting and editorial practices” that was explained as “Constraints and barriers to the practice of reporting relevant detail” with included among the examples “Journal space restrictions” Further, academic publication system hurdles is a separate category at the level of cluster D, science and society and it also includes editors as main actors.

6. After a series of rejections researchers may doubt whether reporting is worth the effort under the pressure of lack of resources such as time. I advise you to read a case study from Helene Speyer - The value of a “failed” trial ^4^.

**Response**: There is a point where efforts to try to publish work are not in balance with the perceived or witnessed chances of it actually being published. This was what we found in the literature as one of the determinants of selective reporting.

*To clarify the basis of the statement, We started the second section of “A framework of possible causal pathways to reporting bias,” with: “ In the review, we found that..”*

Obviously, trials that fail because they do not confirm the hypothesis are as valuable as trials with expected results or results hoped for. We appreciate the article of Speyer, her personal account of initial doubts and efforts to publish the results of a strong trial that in fact delivered strong evidence that the intervention was ineffective. It calls for editors and reviewers to reflect on their own possible biases and focus on the quality of the work rather than judging the quality of the work in light of their perspectives on the results and how the results may or may not differ from previous work.

*We added to the Discussion: The model also shows that for example editors may influence the outcome in multiple ways; first, directly via Means (the category Limitations in reporting and editorial practices). Second, editors as a collective affect the rejection rates through Academic publication system hurdles (the category of Pressures from science and society), but also the extent to which authors find efforts to publish worthwhile (category of cluster Conflicts and balancing of interests).The latter is illustrated by the personal account of Speyer (2018).*

*Other changes.*

*We edited the text at several places, shortening it where possible.*

*In the Discussion, we shortened the example of an intervention with regard to the means for comparing protocols and publication. Software to compare protocol and publication was not necessary in the NIHR Journal Library system and we added reference to Wright et al. (2018) for this*.
